# Oral Administration of an *Opuntia ficus-indica* Fruit Extract Induces Changes in Gut Microbiota Composition: Relationship with Its Anti-Obesity and Anti-Steatotic Effects in Rats Fed a High-Fat High-Fructose Diet

**DOI:** 10.3390/foods14162891

**Published:** 2025-08-20

**Authors:** Iker Gómez-García, Irene Besné-Eseverri, Maria P. Portillo, Alfredo Fernández-Quintela, Ligia Esperanza Díaz, Jose I. Riezu-Boj, Fermín I. Milagro, Jenifer Trepiana

**Affiliations:** 1 Nutrition and Obesity Group, Department of Nutrition and Food Science, Lucio Lascaray Research Institute, University of the Basque Country (UPV/EHU), 01006 Vitoria-Gasteiz, Spain; iker.gomez@ehu.eus (I.G.-G.); irene.besne@ehu.eus (I.B.-E.); alfredo.fernandez@ehu.eus (A.F.-Q.); jenifer.trepiana@ehu.eus (J.T.); 2 Bioaraba Health Research Institute, 01006 Vitoria-Gasteiz, Spain; 3 CIBERobn Physiopathology of Obesity and Nutrition, Institute of Health Carlos III, 28029 Madrid, Spain; fmilagro@unav.es; 4 Immunonutrition Group, Department of Metabolism and Nutrition, Institute of Food Science, Technology and Nutrition (ICTAN), Spanish National Research Council (CSIC), C/Jose Antonio Nováis 6, 28040 Madrid, Spain; ldiaz@ictan.csic.es; 5 Department of Nutrition, Food Sciences and Physiology, Center for Nutrition Research, University of Navarra, 31008 Pamplona, Spain; jiriezu@unav.es; 6 Navarra Institute for Health Research (IdiSNA), 31008 Pamplona, Spain

**Keywords:** obesity, MAFLD, bioactive compounds, microbiota, *Opuntia ficus-indica*, high-fat high-fructose diet

## Abstract

Diseases such as obesity and metabolic-dysfunction-associated fatty liver disease (MAFLD) are often associated with changes in gut microbiota composition. The present study aims to investigate the relationship between the potential preventive effects of an *Opuntia ficus-indica* var. *colorada* cactus pulp extract on obesity and hepatic steatosis, and changes in gut microbiota composition, in a murine model fed a high-fat high-fructose diet. The low-dose extract was the most effective in reducing hepatic TG (−12.5%) and the weight of subcutaneous and visceral adipose tissue (−18.4% and 11.4%, respectively), while the high dose led to improved serum lipid profile (−74.2% in TG, −37.2% in total cholesterol, −50.5% in non-HDL cholesterol and +71.7% in HDL cholesterol). *Opuntia* extract supplementation did not prevent the dysbiosis in gut microbiota produced by the high-fat high-fructose diet. However, modifications in its composition, consistent with an increment in both *Adlercreutzia muris* and *Cutibacterium acnes*, and a reduction in *Massiliimalia timonensis*, were observed. It can be proposed that these changes may contribute to the extract effects against obesity and liver steatosis. Nevertheless, further research is required to establish a direct link between the anti-obesity and anti-steatotic effects and the functionality of the bacteria modified by the treatment.

## 1. Introduction

Recent reports indicate that over half of the global population will become overweight or obese by 2035 if preventive actions are not taken [1]. Obesity is a chronic complex disease defined by excessive fat deposits, associated with higher rates of death driven by co-morbidities such as type 2 diabetes (T2D), dyslipidemia and liver disease [2]. Metabolic-dysfunction-associated fatty liver disease (MAFLD) is a chronic condition characterized by hepatic fat accumulation (≥5% hepatic steatosis) accompanied by one of the following: obesity or overweight, TD2, or evidence of metabolic dysregulation (low-grade inflammation, increased oxidative stress, mitochondrial dysfunction or intestinal dysbiosis) [3,4].

Microbial diversity and richness are key indicators of a healthy gut microbiota. The role of diet in shaping gut microbiota composition is well established. Macronutrients, micronutrients and dietary fiber significantly impact gut microbiota diversity and composition [5]. Intestinal dysbiosis, an imbalance of the microbiota characterized by a reduced diversity, increased abundance of pathogenic bacteria, and a loss of beneficial bacteria, has been described in various pathological conditions. Regarding hepatic diseases, evidence shows that gut microbiota composition may be related to different stages of liver disease [6]. Thus, a dysbiosis has been identified in patients at different stages of MAFLD [7], although discrepancies across studies prevent the definition of a consistent MAFLD gut microbiota profile associated with the condition. A decreased abundance of Bacteroidetes and Ruminococcaceae and an increased abundance of Lactobacillaceae, Veillonellaceae and Dorea are the most frequently reported alterations [8]. In addition, different dietary patterns such as the Western diet, characterized by high fat, high sugar and low fiber intake, are associated with a reduction in bacteria with anti-inflammatory properties (including *Akkermansia muciniphila*, *Faecalibacterium prausnitzii*) and a decreased production of short-chain fatty acids (SCFAs) [9]. Similarly, this type of dietary pattern promotes the growth of microbes that, under dysbiotic conditions, contribute to the development of certain pathologies, potentially resulting in inflammation of the colon. Furthermore, excessive fat intake directly alters gut microbiota composition, promoting the production of pro-inflammatory mediators and impairing intestinal barrier function [9].

Beyond pharmacological treatment, lifestyle modifications remain the first-line strategy for the primary prevention and management of obesity or MAFLD pathogenesis [10]. In addition, health status, dietary habits and lifestyle are considered key determinants shaping the gut microbiota, with diet being one of the most pivotal factors [11]. Several dietary strategies have been linked to inducing weight loss and improving gut microbial diversity and dysbiosis [12]. Certainly, the benefits of a therapeutic dietary approach are increasingly recognized for their role in improving the gut microbial environment [13]. Mutual interactions between gut microbes and undigested nutritional substrates or naturally bioactive compounds (e.g., phenolic compounds) can modulate both the genetic composition and the metabolic functions of intestinal bacteria [13,14].

The genus *Opuntia* is characterized by the production of pear-shaped fruits, commonly known as prickly pears. Belonging to the Cactaceae family, *Opuntia* encompasses over 250 species that grow wild across the Americas, Asia, Africa, Oceania and parts of the Mediterranean region. Interestingly, extracts from opuntioid cacti have been reported to exert beneficial effects in the prevention and treatment of several disorders, including obesity and its metabolic co-morbidities such as liver diseases [15,16]. Therefore, their inclusion in the diet may help ameliorate these pathological conditions. Regardless of the large number of species within the *Opuntia* genus, most studies have focused on *Opuntia ficus-indica*. Although its phytochemical composition varies depending on the part of the plant (e.g., cladodes, fruits/seeds, flowers and roots) [17], the fruit pulp is considered a major source of bioactive compounds, including betalains (betacyanins and betaxanthins), phenolic compounds (flavonoids and phenolic acids) and carotenoids.

Taking all this into account, the aim of the present study is to evaluate the effects of supplementation with an extract rich in bioactive compounds, obtained from the pulp of *Opuntia ficus-indica* var. *colorada* fruit, on microbiota alterations in rats fed a high-fat high-fructose diet, and to establish a potential relationship between these effects and the prevention of obesity and liver steatosis associated with this supplementation.

## 2. Materials and Methods

### 2.1. Opuntia fichus-indica Var. colorada Extract

In a previous investigation conducted by our group, the beneficial properties of various extracts from *Opuntia ficus-indica* fruits (pulp or peel) were evaluated on murine AML-12 hepatocytes [18]. Based on the results gathered, the most efficient extract was obtained from the pulp of *Opuntia ficus-indica* var. *colorada*, which was subsequently selected for the present in vivo research. Prickly pears of *Opuntia ficus-indica* var. *colorada* were collected in Fasnia (28°14′44″ N, 16°26′10″ W; 446 m above sea level) in Tenerife, Canary Islands, Spain [19].

In order to obtain the aqueous extracts of prickly pear rich in betalains and phenolic compounds, freeze-dried tissues were pulverized under reduced light conditions. After that, one gram of tissue was extracted with methanol (Sigma-Aldrich, Waltham, MA, USA)/water (1:1, v:v) by homogenizing the sample with a vortex and an ultrasonic water bath, in ice. After a centrifugation (10 min at 10,000× *g*, 4 °C), the supernatants were collected, and the extraction was repeated two additional times (methanol/water; 1:1, v:v). One last time, samples were extracted with pure methanol, and the supernatants were evaporated in a rotary evaporator at 30 °C until their volumes were reduced [20]. The extracts were freeze-dried and then dissolved in water to prepare stock solutions, filtered, aliquoted and stored at −20 °C until administration to the animals.

As shown in Table 1, the pulp extract of *Opuntia ficus-indica* var. *colorada* is rich in betalains and phenolic compounds, with particularly high levels of indicaxanthin (a betalain) and piscidic acid (a phenolic compound). Other betaxanthins are also present, albeit in smaller quantities.

### 2.2. Animals, Diets and Experimental Design

Forty male Wistar rats (4 weeks old; 125–145 g) were acquired from Envigo (Barcelona, Spain) for the experiment, which was conducted in accordance with the University of the Basque Country’s guidelines for the care and use of laboratory animals (M20_2022_283, 2 March 2023). Rats were housed in pairs in polycarbonate cages in a temperature-controlled room (22 ± 2 °C) with a 12 h light–dark cycle. Following a one-week acclimatization period, the animals were randomly assigned to four experimental groups. The control group (C group; *n* = 10) received a standard commercial diet (D10012G; Research Diets, New Brunswick, NJ, USA), while the remaining groups were fed a high-fat high-fructose (HFHF) diet (D21052401; Research Diets, New Brunswick, NJ, USA; Table 2). Some of the rats in the HFHF diet were supplemented with a daily oral solution containing 2.5% sucrose and the appropriate amount of *Opuntia ficus-indica* var. *colorada* pulp extract to achieve either a low dose (25 mg/kg body weight; L-OFI group; *n* = 10) or a high dose (100 mg/kg body weight; H-OFI group; *n* = 10). The choice of doses used in this in vivo study was based on the available literature, where most of the authors administered doses of *Opuntia* extracts in the range of 25 to 300 mg/kg/d, with the dose of 100 mg/kg/d being the most commonly used [16]. The rats in the control and HFHF groups (*n* = 10 per group), which did not receive the extract, were given only the 2.5% sucrose solution as the vehicle. All treatments and the vehicle were administered daily using a plastic Pasteur pipette. Animals had ad libitum access to food and water, and both food intake and body weight were recorded daily. The treatment period lasted eight weeks.

Rats were fasted for 12 h prior to the conclusion of the experiment. Fecal samples were collected in a tube the day before sacrifice (following overnight fasting) by housing the animals individually and inducing defecation through gentle abdominal massage. The final treatment was administered 3 h before euthanasia, which was performed under anesthesia (chloral hydrate) via cardiac exsanguination. The liver and adipose tissues from various depots were excised, weighed and immediately frozen in liquid nitrogen. Serum was obtained by centrifugation of blood samples (1000× *g*, 10 min and 4 °C). All samples were stored at −80 °C until analysis.

### 2.3. Determination of Liver Triglyceride Content and Serum Biochemical Parameters

Total hepatic lipids were extracted following the procedure outlined by Folch et al. [21]. The lipid extract was subsequently dissolved in isopropanol, and the triglyceride concentration was determined by spectrophotometry using a commercial kit (Spinreact, Barcelona, Spain). Serum concentrations of total cholesterol, non-high-density lipoprotein-cholesterol (non-HDL cholesterol), alanine aminotransferase (ALT) and aspartate aminotransferase (AST) were assessed with commercial kits (BioSystems, Barcelona, Spain). Serum triglyceride content (TG) was measured using a commercial kit (Spinreact, Barcelona, Spain).

### 2.4. Liver Histological Analysis

Immediately after sacrifice, a liver sample from the same lobe of each animal was fixed in 10% buffered formalin and subsequently embedded in paraffin. Liver sections were stained with hematoxylin and eosin using standard techniques. Biopsies were classified into four grades based on fat accumulation according to Brunt et al. [22]: grade 0 indicated no hepatic fat; grade 1, fat vacuoles in less than 33% of hepatocytes; grade 2, fat vacuoles in 33–66% of hepatocytes; and grade 3, fat vacuoles in more than 66% of hepatocytes. Two experienced pathologists, blinded to the experiment groups, independently evaluated all samples and reached a consensus on the classification of each biopsy.

### 2.5. Fecal DNA Extraction and 16S rRNA Gene Amplification for Microbiota Composition Analysis

DNA was extracted from fecal samples using the QIAamp DNA stool MiniKit (QIAGEN, Hilden, Germany) following the manufacturer’s instructions. Microbiota composition was assessed by amplifying the variable V3 and V4 regions of the bacterial 16S ribosomal RNA gene (16S rRNA) from the fecal DNA, followed by sequencing on the Illumina MiSeq platform (Illumina, San Diego, CA, USA), as explained elsewhere [23].

The 16S rRNA gene sequence data were processed using the Quantitative Insights Into Microbial Ecology program (QIIME2) [24]. Low-quality reads were filtered, and subsequently, chimeric sequences were removed. Clean reads were clustered into amplicon sequence variants (ASVs) using DADA2 [25] and annotated with the SILVA v.132 16S rRNA gene reference database [26]. The relative abundance of each ASV was calculated using the Phyloseq R package (version 4.4). Features with fewer than four counts in 80% of the samples were excluded. Microbiota analyses were performed using the MicrobiomeAnalyst 2.0 platform [27], and data were normalized by the centered log ratio (CLR) method [28]. The files were deposited in the NCBI repository, and the BioProject ID is as follows: PRJNA1293542.

### 2.6. Short-Chain Fatty Acid (SCFA) Analysis

An aliquot of 100 mg of frozen fecal sample was diluted in 1 mL of Milli-Q water, followed by homogenization and freezing of the fecal homogenates for dry matter precipitation. Stool samples were then thawed and centrifuged for 15 min at 12,000–13,500 rpm (MPW-150R, med instruments, Warsaw, Poland). The SCFAs acetic, propionic, butyric, isobutyric, valeric and isovaleric acids were quantified in the supernatants by gas chromatography and flame ionization detection (GC-FID, Agilent 6890A, Agilent Technologies, Waldbronn, Germany). The capillary chromatographic column used was a DB-WAXtr column (100% polyethylene glycol, 60 m, 0.325 × 0.25), and helium was used as the carrier gas at 1.5 mL/min. Injection was performed in splitless mode, with an injection volume of 2 μL and a temperature of 250 °C. Methyl valeric was used as an internal standard, and the standard curve was prepared in a similar way to the samples. The detector temperature was 260 °C. The column was heated at 50 °C for 2 min, followed by an increase of 15 °C every min to 150 °C, 5 °C every min to 200 °C, and finally 15 °C every min to 240 °C. The different SCFAs were identified by the retention time of the standard compounds.

### 2.7. Statistical Analysis

Results are expressed as mean ± SEM. Statistical analysis was conducted using SPSS 24.0 (SPSS, Chicago, IL, USA). The normal distribution of the data was confirmed with the Shapiro–Wilk test. One-way ANOVA followed by Newman–Keuls *post hoc* test was applied to compare Chao1 and Shannon indices, body weight, adipose tissue and liver weights, hepatic triglycerides, serum and biochemical parameters and fecal SCFAs among experimental groups. Significance was set at the *p* < 0.05 level.

Regarding microbiota analysis, corrected *p*-values (*q*-values) < 0.05 were considered significant, with adjustments conducted using the Benjamini–Hochberg method. Alpha diversity was estimated with the Chao1 and Shannon indices, and comparisons between groups were performed by Kruskal–Wallis tests. Beta diversity was calculated based on the Bray–Curtis distance matrix, and significance was assessed by PERMANOVA, visualized using non-multidimensional dimension scale (NMDS). Associations between microbiota changes and other parameters were examined using Spearman correlation, with statistical significance defined as *p* < 0.05.

## 3. Results

### 3.1. Body Weight, Adipose Tissue Weights, Liver Weight, Hepatic Triglyceride Content and Serum Parameters

At the end of the experimental period, total body weight was significantly higher in the HFHF group in comparison with control rats (*p* < 0.05). None of the treatments prevented this increase. The high-fat high-fructose diet also led to a significant boost in visceral and subcutaneous adipose tissue weights. Low-dose supplementation significantly prevented the diet-induced gain in both visceral and subcutaneous tissues (*p* < 0.05), whereas this effect was not observed in the H-OFI group (Table 3). Liver weight was also significantly increased by the HFHF diet, but neither low- nor high-dose supplementation altered this parameter.

**Figure 1:**
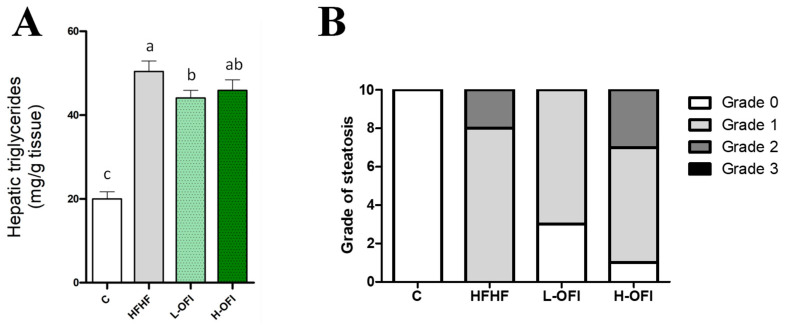
Hepatic triglycerides (**A**) and grade of steatosis (**B**) in rats fed a standard diet (C group) or a high-fat high-fructose diet alone (HFHF group) or supplemented with *Opuntia ficus-indica* pulp extract at a dose of 25 mg/kg weight/day (L-OFI group) or 100 mg/kg weight/day (H-OFI group) for eight weeks. Data not sharing a common letter are significantly different (*p* < 0.05).

**Figure 2:**
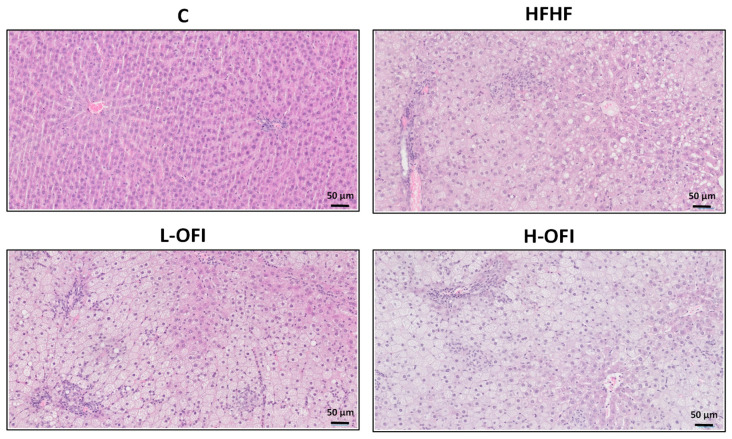
Hepatic histological study using hematoxylin and eosin staining in rats fed a standard diet (C) or a high-fat high-fructose diet alone (HFHF) or supplemented with *Opuntia ficus-indica* pulp extract at a dose of 25 mg/kg weight/day (L-OFI) or 100 mg/kg weight/day (H-OFI). One representative image per group is shown at 200× magnification.

Hepatic triglyceride content was significantly higher in the HFHF group than in controls, indicating the onset of liver steatosis. This increase was significantly attenuated in animals treated with the low dose of the *Opuntia ficus-indica* extract (−12.5%) when compared to the HFHF group (*p* < 0.05), whereas only a modest, non-significant reduction was noted with the high dose (−8.9%) (Figure 1A). Histological analysis confirmed steatosis in the HFHF group (Figure 1B). Consistent with these results, low-dose supplementation decreased the steatosis grade compared to the HFHF group, while a milder effect was observed with the high dose (Figure 1B and Figure 2).

No significant changes in serum triglyceride levels were induced by high-fat high-fructose feeding (Table 4); however, rats treated with the extract at both doses exhibited significantly lower triglyceride concentrations in comparison with the HFHF group (*p* < 0.001). Total cholesterol serum concentrations increased significantly in the HFHF group (*p* < 0.001), an effect completely prevented by the high dose of the extract (*p* < 0.001), with values comparable to those of the control group. In contrast, no significant differences in total cholesterol were observed between L-OFI and HFHF groups. Similarly, non-HDL cholesterol concentrations were elevated in the HFHF group compared to controls (*p* < 0.001), but this increase was significantly prevented by the high dose of the extract (*p* < 0.001), while remaining unchanged (Table 4).

Serum transaminase levels (ALT and AST) were significantly augmented in the HFHF group compared to controls. ALT values remained unchanged by any treatment. In contrast, serum AST was significantly reduced in the H-OFI group when compared to the HFHF cohort (*p* < 0.05), although this decrease did not reach the level recorded in the C group (Table 4).

### 3.2. Microbiota Composition

**Figure 3:**
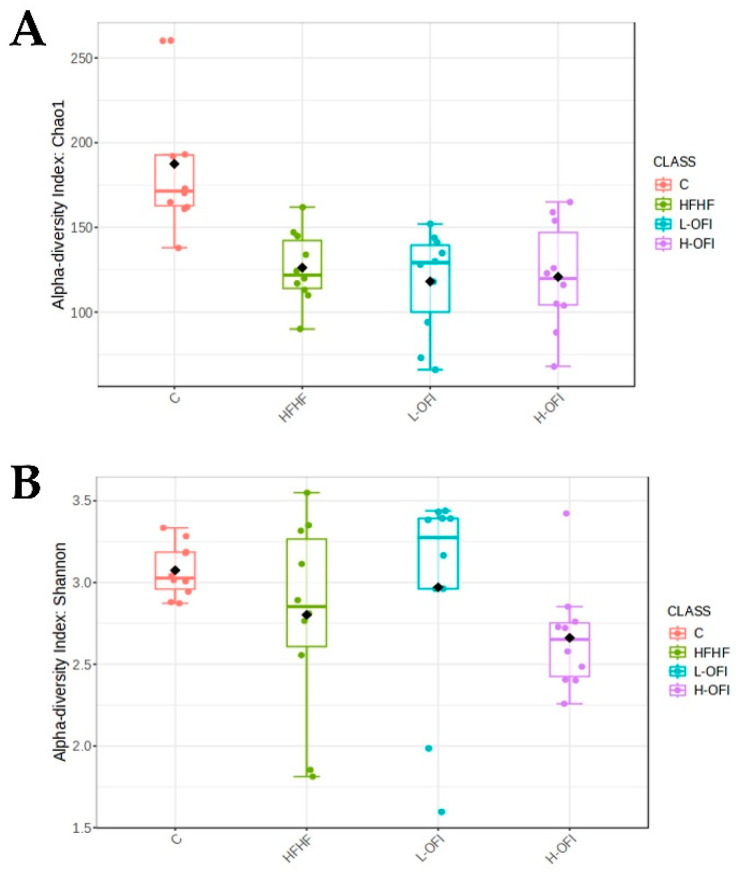
Alpha diversity indices: (**A**) Chao1 and (**B**) Shannon in rats fed a standard diet (C) or a high-fat high-fructose diet alone (HFHF) or supplemented with *Opuntia ficus-indica* pulp extract at a dose of 25 mg/kg weight/day (L-OFI) or 100 mg/kg weight/day (H-OFI) for eight weeks.

Alpha diversity was assessed using Chao1 (richness) and Shannon (diversity) indices. Significant differences were observed between the control and HFHF groups (Chao1 index). Although a slight decrease in the Shannon index was noted for the H-OFI group, no significant differences were seen between the treated groups and the HFHF cohort (Figure 3).

**Figure 4:**
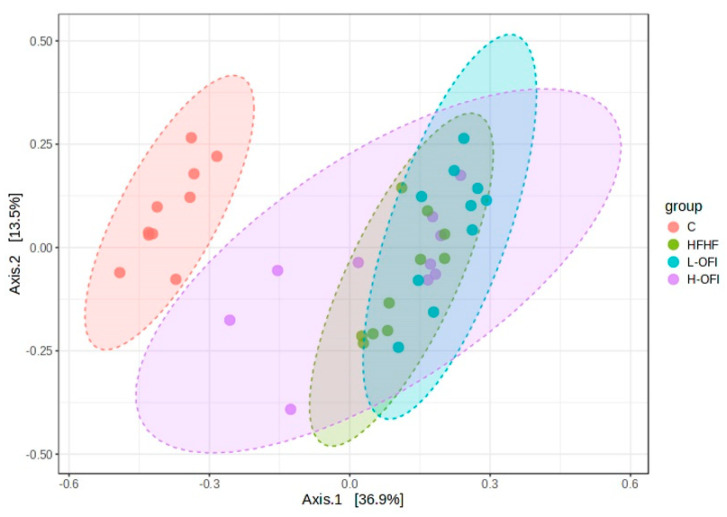
Beta diversity analysis using PCoA of gut microbiota based on Bray–Curtis dissimilarities in rats fed a standard diet (C) or a high-fat high-fructose diet alone (HFHF) or supplemented with *Opuntia ficus-indica* pulp extract at a dose of 25 mg/kg weight/day (L-OFI) or 100 mg/kg weight/day (H-OFI) for eight weeks.

Beta diversity was assessed using Bray–Curtis dissimilarities. As expected, the control group clustered separately from the other groups, with significant differences. Upon removal of the C group, statistical analysis revealed a significant change between the HFHF treatment and the low-dose *Opuntia ficus-indica* extract (R = 0.15, *p* = 0.023). Visual clustering from PCoA was limited, as the first two components explained only a small proportion of the variance. The hierarchical clustering dendrogram indicated slight grouping among the extract-treated groups; however, no clear overall pattern was apparent (Figure 4).

Taxonomic analysis revealed substantial differences between the C and HFHF groups. Given that the primary aim of the present study was to evaluate the potential changes induced by *Opuntia ficus-indica* extracts on gut microbiota composition, only comparisons between the *Opuntia*-treated groups and the HFHF group are presented. In rats receiving the low dose of *Opuntia ficus-indica* extract, significant modulation was observed in one phylum, three classes, two orders, two families, eight genera and four species (Table 5).

In rats treated with the high dose of the *Opuntia ficus-indica* extract, five genera and four species were modified (Table 6).

### 3.3. Fecal Short-Chain Fatty Acid (SCFA) Content

No significant changes were observed in fecal SCFA content. However, a trend towards reduced acetic acid values in H-OFI (*p* = 0.063) and valeric acid in L-OFI (*p* = 0.052) was observed relative to the HFHF group (Table 7).

### 3.4. Correlations Between Microbiota and General Parameters

**Figure 5:**
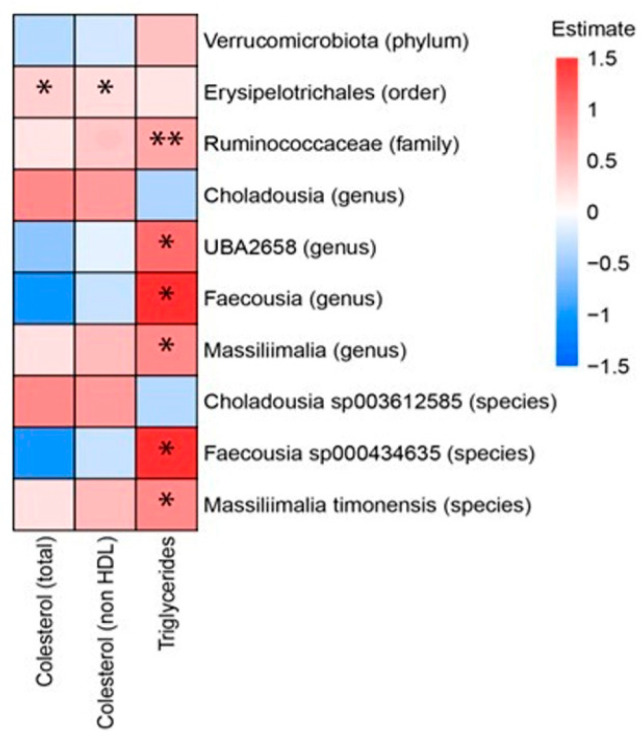
Heat plot showing Spearman’s correlations between microbiota (in rows) and serum parameters (in columns). * *p*-value < 0.5; ** *p*-value < 0.01. The *p*-values were adjusted by FDR (false discovery rate). We have used the combined data of the rats of the three following groups: rats fed a high-fat high-fructose diet alone (HFHF), rats supplemented with *Opuntia ficus-indica* pulp extract at a dose of 25 mg/kg weight/day (L-OFI) and rats supplemented with 100 mg/kg weight/day (H-OFI) for eight weeks.

Among serum parameters, triglycerides exhibited numerous significant correlations. Positive correlations were identified between serum triglycerides and the species *Faecousia sp000434635* and *Massiliimalia timonensis*, as well as with the genera *Massiliimalia*, *Faecousia* and *UBA2658* and the family Ruminococcaceae. The strongest correlations involved the genus *Faecousia* and the species *Faecousia sp000434635*. As for cholesterol, only the order Erysipelotrichales positively correlated with both total cholesterol and non-HDL cholesterol (Figure 5).

## 4. Discussion

In the present study, the administration of a diet rich in fructose and saturated fats resulted in increased body weight, enlargement of all fat pads, and enhanced hepatic triglyceride accumulation, supporting its suitability as a dietary pattern for generating a murine model of both obesity and steatosis. In this regard, only the low dose of the *Opuntia ficus-indica* var. *colorada* pulp extract was effective in reducing triglyceride accumulation in the liver, subcutaneous adiposity and total visceral fat mass in this animal model. Conversely, regarding serum lipids, the high dose was more effective, as it reduced both triglycerides and cholesterol concentrations, whereas the low dose only acted on triglycerides. The anti-obesity and anti-steatotic effects, as well as the reduction in serum lipids, have been previously evidenced with extracts obtained from *Opuntia ficus-indica* cladodes or seeds, and fruit vinegar and juice [15,16]. These preparations differ substantially in the proportions of bioactive compounds compared to the extract used in the present study. To the best of our knowledge, this is the first report to investigate the effects of an extract obtained exclusively from the fruit pulp.

Transaminases are widely accepted as markers of liver damage. Based on this, a reduction in their serum levels would be expected, at least in the L-OFI group. However, no such decrease was observed. Nevertheless, it is important to note that ALT/AST levels are most useful as markers of inflammation or necrosis, rather than direct indicators of liver fat content [29]. The absence of transaminase reductions in the L-OFI group, alongside the decrease in AST in the H-OFI group, may be explained by several factors. If a treatment reduces steatosis but does not improve inflammation or oxidative stress, transaminase levels may remain unchanged or even increase. In this sense, it should be pointed out that in this precise cohort of rats, the low dose of *Opuntia ficus-indica* var. *colorada* pulp extract was less effective in counteracting the oxidative stress and inflammatory response induced by the diet [30]. Furthermore, even after partial fat clearance, the liver may enter a phase of cellular repair, which can sustain elevated transaminase levels. This reflects a temporal disconnect between structural recovery (reduction of fat) and biochemical improvement (liver enzymes) [31].

It is important to note that the “multiple hit” theory, currently employed to explain the progression of MAFLD, identifies gut microbiota dysbiosis as one of the factors contributing to liver damage [32]. Concerning the mechanisms underlying the detrimental effects of an HFHF diet on hepatic function, alterations in gut integrity and increased permeability have been reported, along with inflammation and changes in microbiota composition and metabolite production [33,34]. In this context, *Opuntia* extracts, rich in phenolic compounds and betalains, may serve as complementary agents to enhance the efficacy of current strategies used to manage metabolic disorders like obesity and MAFLD [15,16]. Although the potential beneficial effects of prickly pear extracts in chronic metabolic conditions have been directly attributed to their bioactive components (betalains or phenolic compounds), the possibility that these effects are mediated through gut microbiota modulation cannot be discarded. Therefore, the present study aimed to investigate the impact of a pulp extract from *Opuntia ficus-indica* var. *colorada* on microbiota alterations in rats subjected to a high-fat high-fructose dietary regime, which is known to disrupt gut microbiota composition [35].

Microbial alpha diversity, assessed by Chao1 and Shannon indices, was significantly reduced in animals fed the HFHF diet in comparison with the control group. Similar outcomes have been reported in rodent models exposed to comparable dietary patterns [36,37] and in humans consuming diets with a high-fat content [38,39]. This decline in microbial diversity is considered detrimental, as it reflects a loss in the variety and abundance of beneficial gut microorganisms. A diverse microbial ecosystem is generally more stable and resilient to external disturbances, including antibiotics, infections or dietary shifts. With reduced diversity, the proliferation of pathogenic microorganisms becomes more likely. Moreover, as different bacterial taxa perform specific roles, a decline in diversity may result in the loss of important microbial functions. Finally, diminished richness can contribute to exaggerated or dysregulated immune responses [40]. This alteration was not prevented by the administration of the *Opuntia ficus-indica* var. *colorada* extracts.

At the species level, the low dose of the *Opuntia ficus-indica* extract led to a significant increase in *Adlercreutzia muris*, a member of the Lachnospiraceae family within the phylum Firmicutes, which participates in the fermentation of dietary fibers and the production of SCFAs, when compared to the HFHF group. Nevertheless, this modulation of a single SCFA-producing bacterium does not appear sufficient to alter the overall SCFA profile. The SCFAs produced by *Adlercreutzia muris* and other gut microbes support the intestinal barrier, a key factor in preventing leaky gut syndrome and promoting overall gut integrity. In this context, several studies have reported a decrease in the abundance of the *Adlercreutzia* genus in rodent models of obesity induced by high-fat feeding [41,42,43], as well as in patients with elevated BMI and low physical activity levels [44]. Moreover, a decreased abundance of other *Adlercreutzia* species, such as *Adlercreutzia equolifaciens*, has been observed in patients with liver conditions, including non-alcoholic fatty liver disease (NAFLD), with a progressive decline observed as the disease advances [45]. Accordingly, it can be suggested that the increment in *Adlercreutzia muris* observed in rats supplemented with L-OFI in the present study could contribute, at least in part, to the anti-obesity and hepatoprotective properties of the extract at the low dose.

The gut microbiota in this experimental set was also significantly enriched in *Cutibacterium acnes*. An association between this bacterium and NAFLD has been previously reported, with increased levels described in children and adolescents (7–16 years) diagnosed with NAFLD compared to healthy subjects [46]. Notably, *Cutibacterium acnes* belongs to the phylum Actinobacteriota, whose density has been reported to decrease in patients with obesity [47,48] and in animal models of NAFLD [49]. This aligns with the increased abundance of the Actinobacteriota phylum observed in the microbiota of rats fed the low dose of the *Opuntia ficus-indica* extract. Nevertheless, the functional role of *Cutibacterium acnes* in obesity or NAFLD remains unclear due to the lack of specific evidence to date.

Furthermore, a trend towards lower levels of *Faecousia sp000434635* was found in the L-OFI group relative to HFHF (*p* = 0.0622). Although limited information is available regarding the role of this bacterium in obesity or its co-morbidities, *Faecousia* spp. have been associated with carbohydrate metabolism [50]. This species belongs to the Ruminococcaceae family, and it should be noted that the reduction in Ruminococcaceae abundance induced by the *Opuntia ficus-indica* extract correlated positively with decreased serum triglyceride levels. Nevertheless, further research is required to clarify the metabolic functions and impacts of *Faecousia*.

*Massiliimalia timonensis*, firstly identified in the feces of healthy males [51], was also decreased after treatment with the low dose of the *Opuntia ficus-indica* extract. This species showed a positive correlation with serum triglycerides; however, no further evidence linking it to obesity or MAFLD has been reported to date.

When examining the changes induced by the high dose of the extract, it becomes evident that they differ from those caused by the low dose. The microbiota composition was more markedly influenced by the low dose, which is in line with the significant reduction in liver triglycerides observed in that group, whereas the high dose only showed a trend towards mitigation. The only common alterations in both L-OFI and H-OFI treatments are decreases in the species *Massiliimalia timonensis* and the genera *Massiliimalia_59888* and *UBA2658.* In addition to these changes, the high dose also boosted the abundance of *Paramuribaculum intestinale*, a bacterium belonging to the phylum Bacteroidetes. Not only did the species abundance increase, but the *Paramuribaculum* genus was also more prevalent in rats receiving the H-OFI treatment compared to those on the HFHF diet. It is important to note that information on this bacterium remains limited. In this regard, Fang et al. (2023) investigated the mechanisms underlying the beneficial effects of *Akkermansia muciniphila* on alcoholic liver disease, beyond its known role in improving intestinal barrier function [52]. Their study revealed that *Akkermansia muciniphila* attenuated disease progression during prolonged exposure by modulating host serum metabolism and reshaping the gut microbial community. Notably, this treatment led to increased abundance of *Paramuribaculum intestinale*.

Although the reported literature concerning this topic is scanty, beneficial changes in gut microbiota induced by *Opuntia ficus-indica* extracts, which were correlated with positive effects on anthropometric and biochemical parameters, have been documented in previous studies. Nevertheless, they have used extracts obtained from cladodes, both in animal models [53] and humans [54,55]. Consequently, the composition of the extracts is very different than that of the extract used in the present study. Indeed, whereas cladodes are rich in mucilage, pectin, polysaccharides and flavonoids, fruits are rich in betalains and different phenolic compounds. Although, apart from the present study, no data are available concerning the effects of *Opuntia ficus-indica* fruits on gut microbiota, there is scientific evidence showing that some of the bioactive compounds present in these fruits are able to modulate it. Song et al. (2016) showed that betacyanins provided protection from diet-induced obesity and its related metabolic disorders, an effect that was associated with the modulation of gut microbiota, especially its ability to decrease the ratio of Firmicutes and Bacteroidetes and to increase the relative abundance of *Akkermansia* [56]. On the other hand, indicaxanthin treatment improved the microflora composition by increasing the abundance of healthy bacterial genera, known as producers of short-chain fatty acids, and reducing bacteria related to unhealthy profiles in mice fed a high-fat diet [57]. As far as polyphenols are concerned, a flavonoid extract obtained from *Opuntia ficus-indica* fruits favorably modulated the intestinal microbial community by enhancing the abundance of beneficial bacteria while concomitantly reducing populations of potentially pathogenic bacteria [58]. Moreover, Zhang et al. (2022) proposed that *Opuntia ficus-indica* anthocyanins could change the microbial diversity and flora composition of the mouse gut and promote the production of short-chain fatty acids [59].

An interesting aspect of the present study is that the low dose of *Opuntia ficus-indica* var. *colorada* extract proved to be more effective than the high dose in preventing both obesity and liver steatosis, as well as in modifying gut microbiota composition, an outcome that may seem surprising. Although such a response has not been previously reported for *Opuntia* extracts, a similar pattern was observed in our earlier work on gut microbiota composition changes in rats fed a HFHF diet supplemented with different doses of the phenolic compound pterostilbene [23]. Comparable findings were also reported by Cho et al. (2012) in a study analyzing the effects of two doses of resveratrol on adipose tissue and liver [60]. Indeed, at low doses, bioactive compounds can induce beneficial effects on health, but several phenomena can explain the reduction in their effectiveness with an increase in the dose: (a) they can inhibit essential enzymes or disrupt healthy cellular pathways, (b) they can interfere with the absorption of nutrients or other bioactive compounds, (c) the body may modify their metabolism or excrete them more quickly, (d) many cellular pathways and transporters involved in their beneficial effects can be saturated and (e) some metabolites that are produced in excess from the bioactive compounds may be less active or even toxic.

## 5. Conclusions

In conclusion, the present study demonstrates that the *Opuntia ficus-indica* var. *colorada* extract partially prevents obesity and liver steatosis induced by high-fat high-fructose feeding. In addition, it improves serum lipid profile. Concerning gut microbiota composition, although it is not able to prevent the reduction in alpha diversity induced by high-fat high-fructose feeding, it modulates the composition, mainly at the low dose. Changes in microbiota composition consisted of an increment in both *Adlercreutzia muris* and *Cutibacterium acnes* and a reduction in *Massiliimalia timonensis*. These effects were not accompanied by significant changes in fecal SCFA content, although a trend towards reduced acetic acid and valeric acid was observed with the high dose. It can be proposed that changes in microbiota composition may contribute to the anti-obesity and/or anti-steatotic effects of the extract, but in order to establish a direct link between these effects and the functionality of the bacteria modified by the treatment, further research is required.

## Figures and Tables

**Table 1:** Quantification of the primary phenolic compounds and betalains found in the pulp extract of *Opuntia ficus-indica* var. *colorada* by HPLC.

Compound	Content (µg of Compound/g Dry Weight)
Piscidic acid	2564 ± 108
Indicaxanthin (Bx-proline)	510 ± 14
Isorhamnetin glucoxyl-rhamnosyl-pentoside (IG2)	30.4 ± 3.9
Portulacaxanthin III (Bx-glycine)	30.0 ± 1.6
Vulgaxanthin II (Bx-glutamic acid)	18.0 ± 2.9
Vulgaxanthin III (Bx-asparagine)	14.6 ± 0.9
Quercetin glycoside 2 (QG2)	14.4 ± 3.3
Vulgaxanthin I (Bx-glutamine)	12.4 ± 0.4
Betanin	traces

**Table 2:** Nutritional composition of experimental diets.

	C	HFHF
Total energy (kcal/g)	3.9	4.5
Carbohydrates (energy%)	63.9	40
Fructose (energy%)	-	10
Proteins (energy%)	20.3	20
Lipids (energy%)	15.8	40
Fiber (amount%)	8.6	6.5

C: standard diet; g: grams; HFHF: high-fat high-fructose; kcal: kilocalories.

**Table 3:** Total body weight and the weights of subcutaneous and visceral adipose pads and liver in rats fed a standard diet (C) or a high-fat high-fructose diet (HFHF) either alone or supplemented with *Opuntia ficus-indica* pulp extract at a dose of 25 mg/kg weight/day (L-OFI) or 100 mg/kg weight/day (H-OFI) for eight weeks.

	C	HFHF	L-OFI	H-OFI	ANOVA
Total body weight (g)	425.0± 7.4 ^b^	473.5 ± 10.6 ^a^	467.0 ± 9.0 ^a^	469.0 ± 11.0 ^a^	*p* < 0.05
Visceral WAT weight (g)	30.8 ± 2.8 ^c^	43.7 ± 1.4 ^a^	38.7 ± 2.0 ^b^	40.7 ± 4.1 ^ab^	*p* < 0.05
Subcutaneous WAT weight (g)	13.4 ± 0.7 ^c^	19.0 ± 0.9 ^a^	15.5 ± 1.3 ^bc^	19.9 ± 2.3 ^ab^	*p* < 0.05
Liver weight (g)	10.7 ± 0.3 ^b^	22.0 ± 0.9 ^a^	20.6 ± 1.0 ^a^	21.0 ± 1.1 ^a^	*p* < 0.05

Data not sharing a common letter are significantly different (*p* < 0.05). WAT: white adipose tissue.

**Table 4:** Biochemical serum parameters in rats fed a standard diet (C) or a high-fat high-fructose diet alone (HFHF) or supplemented with *Opuntia ficus-indica* pulp extract at a dose of 25 mg/kg weight/day (L-OFI) or 100 mg/kg weight/day (H-OFI) for eight weeks.

	C	HFHF	L-OFI	H-OFI	ANOVA
Triglycerides (mg/dL)	100.4 ± 2.1 ^a^	93.7 ± 5.1 ^a^	30.1 ± 3.4 ^b^	24.2 ± 2.1 ^b^	*p* < 0.001
Total cholesterol (mg/dL)	63.6 ± 5.4 ^b^	123 ± 4.3 ^a^	132 ± 1.6 ^a^	77.3 ± 5.3 ^b^	*p* < 0.001
HDL cholesterol	13.5 ± 0.7 ^b^	13.1 ± 0.4 ^b^	18.3 ± 1.7 ^a^	22.5 ± 2.0 ^a^	*p* < 0.05
Non-HDL cholesterol (mg/dL)	50.1 ± 5.5 ^b^	109.4 ± 4.6 ^a^	114.4 ± 2.1 ^a^	54.2 ± 6.4 ^b^	*p* < 0.001
ALT (U/L)	14.1 ± 0.7 ^b^	100.1 ± 14.2 ^a^	103.4 ± 13.5 ^a^	97.0 ± 15.1 ^a^	*p* < 0.05
AST (U/L)	75.8 ± 3.7 ^b^	118.2 ± 10.8 ^a^	112.5 ± 3.7 ^a^	93.4 ± 7.0 ^c^	*p* < 0.05

Data not sharing a common letter are significantly different (*p* < 0.05).

**Table 5:** Taxa modulated by the low dose of *Opuntia ficus-indica* extract when compared with the HFHF group.

	Log2FC	St. Error	*p*-Value	FDR
SPECIES				
***Choladousia sp003612585***	**2.35**	**0.62**	**0.000353**	**0.00267**
***Cutibacterium acnes***	**2.58**	**0.836**	**0.00309**	**0.0173**
***Adlercreutzia muris***	**2.14**	**0.797**	**0.00955**	**0.0448**
***Massiliimalia timonensis***	**−1.9**	**0.715**	**0.01**	**0.0458**
*Akkermansia_muciniphila_D_776786*	−2.31	0.89	0.0117	0.0526
*Faecousia_sp000434635*	−4.15	1.65	0.0148	0.0622
GENUS				
***g__UBA2658***	**−3.88**	**0.861**	**0.0000314**	**0.000356**
***g__Choladousia***	**2.35**	**0.62**	**0.000353**	**0.00284**
***g__Cutibacterium***	**2.58**	**0.836**	**0.00309**	**0.0174**
***g__QWKK01***	**2.35**	**0.822**	**0.00591**	**0.0298**
***g__Eubacterium_R***	**−5.4**	**1.92**	**0.00683**	**0.0341**
***g__Adlercreutzia_404257***	**2.22**	**0.804**	**0.00767**	**0.0375**
***g__Massiliimalia_59888***	**−1.9**	**0.715**	**0.01**	**0.0455**
***g__Evtepia***	**−3.04**	**1.15**	**0.0102**	**0.0461**
*g__Akkermansia*	−2.31	0.89	0.0117	0.0521
*g__Adlercreutzia_404199*	2.05	0.79	0.0121	0.053
*g__UBA1367*	2.13	0.842	0.014	0.0601
*g__Faecousia*	−4.15	1.65	0.0148	0.0622
*g__Anaerofilum_74150*	2	0.835	0.0199	0.0778
FAMILY				
**f__Propionibacteriaceae**	**2.58**	**0.836**	**0.00309**	**0.0178**
**f__Eggerthellaceae**	**1.97**	**0.671**	**0.00474**	**0.0252**
f__Akkermansiaceae	−2.31	0.89	0.0117	0.0555
f__Atopobiaceae	2.13	0.842	0.014	0.0642
f__Oscillospiraceae_88309	−2.55	1.01	0.014	0.0642
ORDER				
**o__Propionibacteriales**	**2.58**	**0.836**	**0.00309**	**0.0173**
**o__Coriobacteriales**	**1.96**	**0.656**	**0.00413**	**0.0211**
o__Verrucomicrobiales	−2.31	0.89	0.0117	0.0523
o__Oscillospirales	−2.48	0.963	0.0125	0.0546
CLASS				
**c__Actinomycetia**	**3**	**0.955**	**0.00264**	**0.0136**
**c__Coriobacteriia**	**1.96**	**0.656**	**0.00413**	**0.0186**
**c__Verrucomicrobiae**	**−2.31**	**0.89**	**0.0117**	**0.0444**
PHYLUM				
**p__Actinobacteriota**	**2.01**	**0.624**	**0.00211**	**0.0127**
p__Verrucomicrobiota	−2.31	0.89	0.0117	0.0633

A positive estimate indicates greater abundance in the corresponding treatment compared to the HFHF group, whereas a negative estimate indicates the opposite. Bold text is used when FDR < 0.05. FDR: false discovery rate.

**Table 6:** Taxa modified by the high dose of *Opuntia ficus-indica* extract when compared with the HFHF group.

	Log2FC	St. Error	*p*-Value	FDR
SPECIES				
***Massiliimalia timonensis***	**−2.63**	**0.696**	**0.000371**	**0.00352**
***Bittarella massiliensis***	**−2.8**	**0.762**	**0.000509**	**0.00462**
***Adlercreutzia caecicola***	**1.16**	**0.393**	**0.00448**	**0.0295**
***Paramuribaculum intestinale***	**1.35**	**0.473**	**0.00588**	**0.0345**
*Ventrisoma faecale*	−1.83	0.776	0.0216	0.0967
GENUS				
***g__Massiliimalia_59888***	**−2.63**	**0.696**	**0.000371**	**0.0041**
***g__Bittarella***	**−2.8**	**0.762**	**0.000509**	**0.00532**
***g__Adlercreutzia_404218***	**1.16**	**0.393**	**0.00448**	**0.0287**
***g__Paramuribaculum***	**1.35**	**0.473**	**0.00588**	**0.0344**
***g__UBA2658***	**−2.27**	**0.838**	**0.0089**	**0.0478**
*g__Muricomes_149725*	−1.88	0.706	0.00997	0.0506
*g__Ventrisoma*	−1.83	0.776	0.0216	0.0921
FAMILY				
f__Ruminococcaceae	−1.98	0.728	0.00873	0.0568

A positive estimate indicates greater abundance in the corresponding treatment compared to HFHF group, whereas a negative estimate indicates the opposite. Bold text is used when FDR < 0.05. FDR: false discovery rate.

**Table 7:** Fecal SCFA content in rats fed a high-fat high-fructose diet alone (HFHF) or supplemented with *Opuntia ficus-indica* pulp extract at a dose of 25 mg/kg weight/day (L-OFI) or 100 mg/kg weight/day (H-OFI) for eight weeks.

SCFA (µmol SCFA/g Feces)	HFHF	L-OFI	H-OFI	ANOVA
Acetic acid	12.03 ± 1.59 ^a^	11.87 ± 1.40 ^a^	9.20 ± 0.63 ^a^	NS
Propionic acid	1.69 ± 0.34 ^a^	1.51 ± 0.27 ^a^	1.99 ± 0.28 ^a^	NS
Isobutyric acid	0.42 ± 0.04 ^a^	0.36 ± 0.02 ^a^	0.44 ± 0.04 ^a^	NS
Butyric acid	1.01 ± 0.13 ^a^	0.82 ± 0.17 ^a^	1.01 ± 0.11 ^a^	NS
Isovaleric acid	0.50 ± 0.03 ^a^	0.54 ± 0.03 ^a^	0.49 ± 0.04 ^a^	NS
Valeric acid	0.55 ± 0.12 ^a^	0.30 ± 0.05 ^a^	0.43 ± 0.10 ^a^	NS

Data not sharing a common letter are significantly different (*p* < 0.05).

## Data Availability

The raw data supporting the conclusions of this article will be made available by the authors on request.
